# Omics landscapes of hepatic echinococcosis: bulk foundations, emerging single-cell studies, and analytical considerations

**DOI:** 10.3389/fimmu.2026.1820459

**Published:** 2026-06-15

**Authors:** Qianwen Wang, Xiaopeng Wang, Honglin Yan, Jingping Yuan

**Affiliations:** 1 Department of Pathology, Renmin Hospital of Wuhan University, Wuhan, China; 2 Department of Laboratory Medicine, Peking Union Medical College Hospital, Chinese Academy of Medical Sciences & Peking Union Medical College, Beijing, China

**Keywords:** *Alveolar echinococcosis*, Cystic echinococcosis, immune microenvironment, single-cell RNA sequencing, spatial transcriptomics

## Abstract

Hepatic echinococcosis (HE) is a major zoonotic disease in endemic regions, caused predominantly by *Echinococcus granulosus* sensu lato (cystic echinococcosis, CE) and *Echinococcus multilocularis* (alveolar echinococcosis, AE). Lesion progression reflects a prolonged host–parasite stalemate in which immunoregulation converges with angiogenesis and fibrovascular remodeling to enable chronic persistence, yet the cellular drivers and niche-specific interactions that sustain these lesions remain incompletely defined. While bulk omics has established a valuable foundation by delineating global perturbations in immune pathways, vascular programs, and extracellular matrix remodeling, these approaches average signals across heterogeneous lesions and adjacent liver, limiting the resolution of discrete cell states and intercellular communication that underpin persistence. Recent advances in single-cell RNA sequencing (scRNA-seq) and emerging spatial transcriptomics (ST) are beginning to overcome these limitations by enabling cell-resolved, niche-aware profiling of HE tissues. Early applications in HE have implicated late-stage expansion of SPP1^+^ macrophages, exhausted T-cell programs, and pro-angiogenic myeloid–endothelial crosstalk, providing a more mechanistic and spatially grounded view of fibrovascular remodeling and chronic inflammation. In this review, we synthesize key insights from scRNA-seq and ST studies of HE lesions and adjacent liver, discuss analytical considerations that are particularly relevant to fibrotic and necrotic tissues, and emphasize stage-aware and lesion-zone–aware interpretation. Taken together, we propose an integrative framework that links cell-state diversity to spatial context to prioritize actionable pathways and guide next-generation multi-omic investigations of HE.

## 1 Introduction

Hepatic echinococcosis is a neglected zoonosis caused predominantly by Echinococcus granulosus sensu lato, the agent of cystic echinococcosis (CE), and Echinococcus multilocularis, the agent of alveolar echinococcosis (AE), and is sustained by predator–prey transmission cycles. Adult tapeworms reside in the intestines of definitive hosts, primarily dogs in the case of cystic echinococcosis and foxes or dogs in alveolar echinococcosis, from which eggs are released into the environment. Intermediate hosts, which are predominantly ungulates (e.g., sheep, goats, cattle, and horses) for cystic echinococcosis and small rodents for alveolar echinococcosis, ingest these eggs and subsequently develop metacestode larvae. Humans are accidental, dead-end intermediate hosts via contaminated food or direct contact with infected canids. After ingestion, oncospheres penetrate the intestinal wall and enter the portal circulation. The liver is the main site of establishment, accounting for approximately 65% of cases, followed by the lungs at approximately 25%, while other organs are involved less frequently (1). Hepatic lesions may remain clinically silent for years. Transmission is shaped by ecology, animal husbandry, and socioeconomic factors; therefore, HE control is intrinsically a One Health problem spanning human health, veterinary practice, and environmental management (2–4). Although both forms colonize the liver, CE and AE differ in growth architecture and clinical management. CE typically forms a well-demarcated, fluid-filled cyst surrounded by a host-derived fibrotic pericyst, reflecting an expansile pattern of growth. By contrast, AE displays infiltrative, tumor-like expansion with microvesicles invading parenchyma, vessels, and bile ducts, often without a complete capsule. Accordingly, CE management is guided by imaging-based stage classification and may include watch and wait, percutaneous interventions, surgery, and benzimidazoles, whereas AE more often requires radical resection when feasible and prolonged antiparasitic therapy (5–7). Importantly, both diseases exhibit stage transitions: early lesions are relatively inflammatory and plastic, whereas mature lesions show architectural stabilization driven by fibrovascular remodeling.

Clinically, most infections remain asymptomatic for prolonged periods, and manifestations are largely determined by the site, size, and condition of the cysts. Hepatic involvement may present with hepatomegaly, right upper-quadrant or epigastric pain, nausea, and vomiting, whereas mass effect can produce biliary obstruction, jaundice, or portal hypertension. Cyst rupture or leakage may trigger hypersensitivity reactions ranging from urticaria to, rarely, fatal anaphylaxis, and may seed secondary echinococcosis. In pulmonary disease, rupture into the bronchial tree can lead to expectoration of cyst membranes or to retained material that serves as a nidus for bacterial or fungal superinfection (1, 8).

Traditional bulk omics have provided an important foundation by outlining global pathway perturbations in immunity, hypoxia, extracellular matrix remodeling, and vascular programs in HE (9–11). However, bulk profiling inherently averages signals across mixed cell populations and is particularly vulnerable to composition bias in lesions containing variable proportions of infiltrating immune cells, activated fibroblasts, endothelial cells, hepatocytes, and necrotic debris. As a result, bulk signatures often cannot pinpoint which specific cell states drive persistence, nor can they robustly reconstruct ligand–receptor communication or zone-restricted programs at the parasite–host interface.

Recent advances in single-cell RNA sequencing (scRNA-seq) and emerging spatial transcriptomics (ST) begin to overcome these limitations by enabling cell-resolved, niche-aware profiling of HE lesions and adjacent liver. scRNA-seq can decode the immune and stromal cell composition within lesions and nominate regulatory programs, while ST preserves tissue architecture and allows transcriptional programs to be mapped onto lesion zones and interfaces. Accordingly, in this Review we integrate evidence from traditional bulk omics and recent single-cell and spatial studies to synthesize a stage-aware, lesion-zone–aware framework for interpreting HE pathobiology, and we highlight key analytical priorities and translational directions for next-generation, multimodal profiling of fibrotic and necrotic lesions.

## 2 The traditional omics era: milestones from global landscapes to mechanistic hypotheses

Before the advent of single-cell technologies, our understanding of the immune microenvironment in HE was largely built on conventional bulk omics platforms, including microarrays and bulk RNA sequencing, complemented by proteomics and non-coding RNA profiling (9–12). These foundational studies defined the macroscopic “climate” of host immune responses and nominated key signaling programs underlying the transition from acute inflammation to chronic fibro-inflammatory remodeling.

Time-course transcriptomic studies in murine hepatic infection models have provided essential evidence for understanding HE progression. Lin et al. (13) performed genome-wide expression profiling and delineated distinct transcriptional phases in the infected liver: an early phase dominated by innate immune activation and inflammatory responses, followed by a chronic phase characterized by metabolic reprogramming and progressive fibrogenesis. This progression parallels a canonical Th1-to-Th2/Treg immune deviation, shifting from cytotoxic, interferon-γ (IFN-γ)-centered responses toward tolerance-associated programs driven by interleukin-10 (IL-10) and transforming growth factor-β (TGF-β), a transition widely considered central to immune evasion and long-term parasite persistence (12). Notably, such immune deviation is not unique to HE. In other chronic liver diseases, including cirrhosis, a comparable inflammation–fibrosis axis can be sustained through coordinated crosstalk between macrophages and T cells (14), ultimately maintaining hepatic stellate cell activation and extracellular matrix deposition (15). A conceptually similar macrophage–T-cell axis also operates in schistosomiasis, where egg-induced Th2 responses orchestrate granuloma formation and progressive fibrosis through tightly coupled macrophage and CD4^+^ T-cell programs (16), underscoring that fibro-inflammatory remodeling is a shared outcome of persistent hepatic antigenic stimulation across Platyhelminthes.

As concepts of gene regulation have matured, increasing attention has been directed toward the role of non-coding RNAs (17–20). Using lncRNA–mRNA co-expression networks, Nian et al. (12) implicated non-coding RNA programs in antigen processing, cytokine signaling, and pro-fibrotic transcriptional regulation. Differentially expressed lncRNAs were proposed to act in trans on key transcription factors such as SMAD3 and STAT1, thereby shaping Th17 differentiation and the TGF-β/Smad fibrogenic cascade. Complementing this layer, miRNA profiling has begun to resolve post-transcriptional regulation during infection. Boubaker et al. reported that, one month after E. multilocularis egg infection in mice, hepatic miR-148a-3p was markedly downregulated (~8-fold), accompanied by upregulation of predicted target genes including Vegfa and Hif1a, suggesting that early infection may relieve miRNA-mediated repression of hypoxia-associated and pro-angiogenic programs that support parasite metabolic demands (21). Beyond the liver-tissue compartment, miRNA studies in Echinococcus have profiled infection-responsive signatures in peritoneal macrophages and nominated candidate circulating miRNA biomarkers for alveolar and cystic echinococcosis (22–24). Pathological vascular remodeling is also a defining feature of tumor microenvironments (25, 26), raising the possibility that the “tumor-like” invasive phenotype of AE may share convergent molecular logics at the level of angiogenic programming (27).

Beyond transcriptomic approaches, proteomic profiling of hydatid cyst fluid (HCF) and parasite tissues has revealed a diverse array of molecular constituents, including well-characterized parasite antigens such as AgB and Ag5, alongside numerous host-derived proteins (28). These discoveries not only help explain cyst-associated hypersensitivity and immune complex deposition but also provide an antigenic basis for serological diagnosis. In biomarker discovery, circulating small RNA sequencing (29) has detected parasite-derived small RNAs (e.g., emu-miR-87-3p) in the serum of patients with alveolar and cystic echinococcosis, demonstrating that Echinococcus genetic material is released into the host circulation, together with host-response–associated small RNAs linked to fibrosis; these have been proposed as candidate noninvasive indicators of disease. However, a persistent unresolved issue is whether such circulating signals originate predominantly from parasite-derived extracellular vesicles or reflect hepatocyte injury and leakage from the host, complicating biological interpretation and clinical specificity.

Whereas many earlier studies emphasized host responses, Herz et al. (30) recently redirected attention to parasite-intrinsic mechanisms. Through transcriptome profiling of E. multilocularis larvae and germinative cell cultures, the authors identified “stem-like” programs that may underlie sustained parasite growth and regeneration. Germinative cells function as a renewable proliferative source, conceptually analogous to tumor-initiating cells, and their selectively expressed pathways nominate potential therapeutic vulnerabilities. Together with earlier parasite genome resources (31, 32), these findings support a dual perspective: the host constructs a fibrotic barrier to contain the parasite, while the parasite mobilizes specialized metabolic enzymes and signaling programs to survive and expand under sustained immune pressure (33).

**Figure 1:**
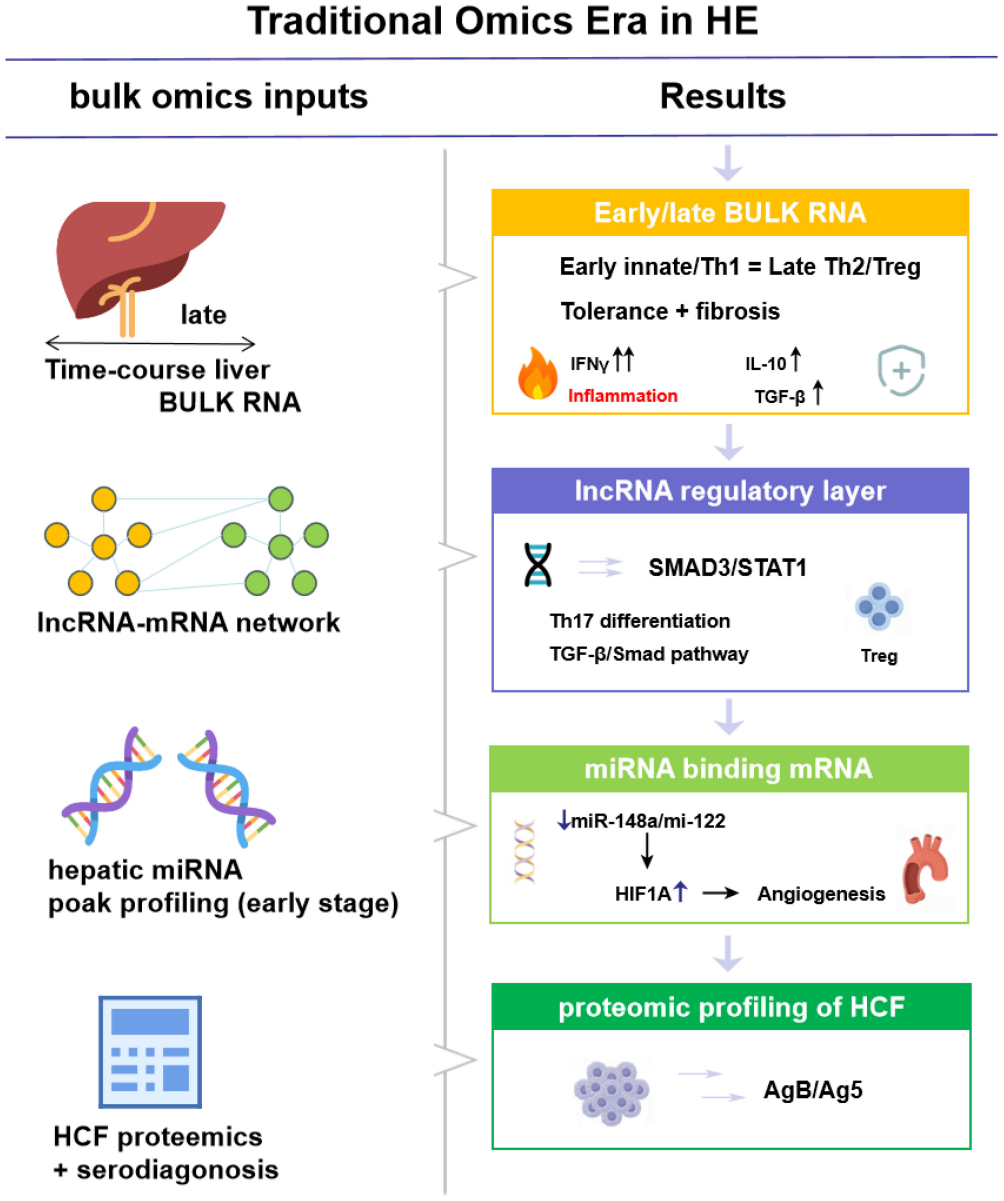
Conceptual schematic of findings in the traditional bulk-omics era of hepatic echinococcosis. Schematic summary of representative bulk-omics inputs (left) and the major biological inferences they enabled (right) during the pre–single-cell era. Time-course liver bulk RNA profiling captured stage-associated immune remodeling, with an early Th1-biased inflammatory phase transitioning toward a late Treg-skewed, tolerance-associated program accompanied by fibrovascular remodeling. Bulk lncRNA–mRNA network analyses highlighted an additional regulatory layer implicating SMAD3/STAT1-linked pathways and T-cell differentiation modules. Early-stage hepatic miRNA profiling suggested miRNA-mediated control of angiogenesis pathways, including reduced miRNA with increased HIF1A signaling and pro-angiogenic outputs. Proteomic profiling of hydatid cyst fluid, often integrated with serodiagnosis, prioritized dominant parasite antigens as biomarkers and diagnostic targets. HE, hepatic echinococcosis; HCF, hydatid cyst fluid; lncRNA, long non-coding RNA; miRNA, microRNA; ST, spatial transcriptomics.

Despite their foundational contributions (**Figure 1**), the central limitation of traditional omics lies in tissue homogenization. Pro-inflammatory M1 macrophages, pro-fibrotic M2 macrophages (34, 35), activated fibroblasts, and injured hepatocytes coexist within the same lesion ecosystem. Consequently, a bulk signal such as elevated TGF-β cannot be unambiguously assigned to infiltrating Tregs, remodeled macrophage states, activated stromal populations, or parasite-associated material. This ambiguity is intrinsic to bulk assays. In line with human liver single-cell atlases (36, 37), the hepatic immune landscape is highly heterogeneous, and rare yet functionally dominant subsets can be masked by transcriptional mass from abundant populations. Therefore, resolving the cellular interaction networks that govern HE lesion persistence and remodeling increasingly requires cell-resolved approaches.

To contextualize these omics-derived inferences, it is useful to summarize the immune response they collectively describe. Following hepatic establishment, an initial innate and Th1-polarized response—dominated by neutrophils, monocytes/macrophages, and IFN-γ—attempts parasite killing, but with lesion maturation the response characteristically deviates toward a Th2 and regulatory profile marked by IL-4/IL-5/IL-13, IL-10 and TGF-β, eosinophilia, IgE and IgG4, and expansion of regulatory T cells (8, 27). Parasite-derived antigens such as AgB modulate dendritic-cell maturation and skew T-cell differentiation toward this tolerogenic state, while alternatively activated (M2) macrophages promote fibrosis and angiogenesis. This Th1-to-Th2/Treg transition is widely regarded as central to immune evasion and the chronic fibro-inflammatory remodeling that defines established HE, and provides the immunological backdrop against which the cell-resolved findings discussed below should be interpreted (38, 39).

## 3 scRNA-seq: historical context and analytical workflow

scRNA-seq has emerged as a transformative technology that enables transcriptomic analysis at single-cell resolution, directly addressing the “averaging” limitation of traditional bulk assays. The field was pioneered in 2009 by Tang et al., who first demonstrated the feasibility of profiling the whole transcriptome of a single mouse blastomere (40). Early plate-based protocols, however, were constrained by limited throughput and high per-cell cost. The paradigm shifted around 2015 with the advent of droplet-based microfluidics, enabling parallel encapsulation and barcoding of thousands of cells in a single run (41, 42). The subsequent maturation and wide adoption of commercialized platforms—exemplified by the 10x Genomics barcoding chemistry and massively parallel digital transcriptional profiling—further democratized high-throughput scRNA-seq and accelerated atlas-scale profiling across tissues and diseases (43). Collectively, this “resolution revolution” has enabled fine-grained cell subtyping, reconstruction of differentiation or activation continua, and network-level inference of intercellular and transcriptional regulation, providing an essential framework to dissect complex pathogenic microenvironments such as hepatic echinococcosis.

### 3.1 From fibrotic lesion to count matrix: upstream considerations

Hepatic echinococcosis lesions present distinct technical challenges for single-cell profiling. Dense pericystic fibrosis often resists enzymatic dissociation, while necrotic regions may release substantial cell-free RNA. Additionally, cyst fluid components can contribute ambient transcripts, collectively increasing the risk of biased cellular recovery and spurious marker expression in downstream analysis. Accordingly, enzymatic digestion and mechanical disruption must be tuned to preserve fragile parenchymal populations while avoiding selective loss of stromal and vascular subsets embedded in the fibrotic rim. To ensure methodological transparency and cross-study comparability, comprehensive reporting must detail the sampling region, lesion stage, prior treatment history, and, in experimental models, the precise duration of infection. After sequencing, alignment to the host reference genome yields the cell-by-gene count matrix. In HE-focused single-cell studies, upstream variables are not merely technical details; they can dominate downstream clustering and “cell state” inference if not controlled, particularly when comparing time points or cohorts (36, 38). Parallel strategies may quantify parasite-derived reads, but stringent filtering is required to mitigate misassignment and to control ambient contamination introduced by cyst fluid. In practice, parasite-read claims are most credible when supported by conservative mapping thresholds and robustness checks.

### 3.2 Primary analysis: QC, integration, and annotation

Quality control (QC) typically removes low-complexity barcodes and cells with high mitochondrial fractions, consistent with stressed or dying cells. In HE, ambient RNA contamination from necrosis or cyst fluid can be substantial and may create misleading “hybrid” signatures if not corrected. Ambient-RNA removal and doublet detection are therefore especially important in HE to reduce marker leakage and eliminate spurious mixed clusters (44–46). When integrating multi-batch data, batch correction approaches such as Seurat integration or Harmony are commonly applied to preserve biological variance while minimizing technical effects (47, 48). An HE-specific best practice is to explicitly test post-correction contamination: after ambient correction and integration, assess whether perilesional hepatocyte markers remain enriched in immune clusters; if so, apply stricter QC or ambient removal and re-evaluate cluster stability. Cell type annotation should be based on both established marker genes and reference atlas data. Whenever possible, such annotations ought to be corroborated through orthogonal techniques like immunostaining or flow cytometry. This validation is especially critical for confirming cellular enrichment patterns at the lesion edge, a histological feature central to the pathogenesis of hepatic echinococcosis (49).

### 3.3 Downstream inference: trajectories, communication, and regulatory networks

Beyond cell typing, scRNA-seq supports inference of dynamic processes. Trajectory methods and RNA velocity can model continua such as infiltrating monocytes differentiating into remodeling macrophages, or fibroblast activation across infection stages (50–52). In HE, trajectories are most interpretable when anchored to explicit biological axes, such as infection duration in mouse models or lesion-zone stratification, so that spatial gradients are not conflated with temporal activation programs. Ligand-receptor inference tools (53–57) can nominate candidate intercellular signaling axes, such as myeloid-endothelial angiogenic programs or myeloid-fibroblast fibrogenic loops but these results should be treated as hypotheses requiring spatial or functional validation (53, 58). Gene regulatory network (GRN) inference can identify master transcription factor regulons stabilizing pathological states (59, 60), offering entry points for mechanistic perturbation and therapeutic targeting (61).

## 4 Single-cell discoveries in HE: immune and stromal ecosystems

Macrophages emerge as a dominant immune compartment across HE datasets, consistent with their roles in parasite containment and tissue remodeling (62–64). In a mouse CE liver scRNA-seq atlas profiling ~45,199 cells at 1, 3, and 6 months post-infection, an Spp1^+^ macrophage state expanded prominently at late stages and was scarce in early infection and uninfected controls (36). SCENIC-based regulatory analysis nominated Cebpe, Runx3, and Rora as candidate transcription factors of this state, and cell–cell communication analysis identified Spp1^+^ macrophage–endothelial signaling via Vegfa–Vegfr1/Vegfr2, linking these macrophages to angiogenic remodeling (36). In human CE lesions, single-cell profiling of 81,865 immune cells likewise revealed a perilesional shift toward immunosuppression, with relative enrichment of Treg-CD4^+^ and Th2-CD4^+^ cells, ILC2s, and pDCs and upregulation of inhibitory programs including the NKG2A/HLA-E checkpoint (38). Mechanistically, the relevant unit of pathogenesis is likely a stable remodeling state rather than a single marker: Spp1^+^ macrophages can co-express extracellular-matrix regulators, checkpoint ligands, and angiogenic factors that together create a permissive niche. Conceptually, these macrophages resemble scar-associated macrophages described in chronic human liver injury (49, 65), implying that parasite persistence may exploit conserved wound-healing circuits. A key unresolved question is whether Spp1^+^ macrophages represent a terminal endpoint or a reversible state. If they arise along a monocyte-to-macrophage trajectory, there may be a stage-defined window in which incoming monocytes can be redirected away from remodeling fate, motivating time-resolved single-cell profiling coupled with perturbation strategies.

DCs are professional antigen-presenting cells that orchestrate T-cell priming against parasites; however, the nature of the ensuing response is highly context-dependent, and in chronic helminth infection the balance among Th1, Th2, and potentially pathogenic Th17 programs is not uniformly protective (8, 27). Chronic HE often exhibits an apparent “presentation–execution gap”: antigen-presentation programs are detectable, but sterilizing immunity fails. Human hepatic CE scRNA-seq demonstrates substantial immune heterogeneity in lesion microenvironments and supports the idea that parasite-adjacent zones may be immunologically constrained (38). A recent AE-focused study profiling hepatic DC subsets in patients and a mouse model found that conventional (cDC) and plasmacytoid (pDC) DCs were enriched in perilesional liver relative to the periphery, and that cDCs upregulated the co-stimulatory molecule CD86 together with immune-checkpoint molecules (CD244, LAG3, CTLA4) and the checkpoint ligands CD48, PD-L1, and CD155; in the mouse model, DCs displayed a tolerogenic phenotype early (reduced CD40/CD80) that shifted toward activation at later stages, indicating spatial and temporal bottlenecks where DCs are present but functionally skewed toward impairing T-cell responses (39). Two non-mutually exclusive mechanisms could contribute: parasite-derived excretory/secretory products may impose tolerogenic programming on DCs, and lesion architecture—fibrosis, altered vasculature, hypoxia—may restrict DC migration and productive T-cell priming. These hypotheses strengthen the rationale for integrating scRNA-seq with spatial methods and functional assays.

Adaptive immunity in HE reflects a balance between containment and tolerance. Lesion-associated T cells may show checkpoint expression and diminished effector programs, consistent with persistent antigen exposure and immunoregulatory cues (38). Complementary mouse AE single-cell work has further resolved stage-dependent transcriptional reprogramming of hepatocytes at the lesion interface, underscoring that parenchymal cells are active participants rather than bystanders (66). Importantly, single-cell immune-repertoire profiling suggests that HE is not solely a local hepatic phenomenon. In mouse infection, a dataset combining scRNA-seq with single-cell TCR/BCR sequencing of spleen and PBMCs reported broad immune remodeling and distinct alterations in Treg subpopulations at late stages of E. granulosus infection (67). These systemic changes support a model of peripheral imprinting, in which chronic hepatic lesions shape circulating immune states that may be leveraged for monitoring.

While macrophage-centric narratives dominate, early infection is likely influenced by neutrophils and inflammatory monocytes that can mediate parasite damage and initiate local inflammation (68, 69). Spatiotemporal multi-omics work in AE integrating scRNA-seq and spatial transcriptomics(ST) indicates heterogeneous neutrophil and macrophage subpopulations with distinct roles in parasite-killing versus later immunosuppression (70). This supports a temporal switch model: early myeloid inflammation may contribute to containment, whereas persistent stimulation may drift toward remodeling and tolerance. Translationally, this argues against non-specific suppression of myeloid activity in early disease and instead favors stage-aware intervention strategies.

Angiogenesis and fibrosis are hallmarks of chronic HE. Single-cell analyses in CE mouse livers highlight myeloid–endothelial communication and Vegfa-associated signaling consistent with angiogenic remodeling near lesions (36). However, standard 3′ scRNA-seq generally cannot resolve VEGFA transcript isoforms reliably; isoform-specific conclusions require isoform-aware bulk RNA-seq, long-read sequencing, or targeted assays. Concurrently, fibroblasts or stellate cells (71) produce collagen (COL1A1/COL3A1) and organize the fibrotic rim. Here, the rim should not be viewed as a passive wall; it likely functions as an active immunological filter that shapes infiltration, establishes chemokine gradients, and creates a diffusion barrier that may contribute to incomplete benzimidazole exposure and treatment responses. Stromal heterogeneity remains under-resolved in HE, and future studies will benefit from stromal enrichment, nuclei-based approaches (snRNA-seq) to mitigate dissociation bias, and spatial validation linking stromal states to collagen architecture.

Host-centric single-cell studies typically capture downstream consequences of parasite influence but rarely quantify parasite “inputs” directly. Parasite excretory products, including small extracellular vesicles (sEVs), are increasingly recognized as active modulators of host immunity (72, 73). One CE mouse liver infection resource explicitly frames immune remodeling in relation to E. granulosus-derived sEVs, enabling hypothesis testing of which host cell states most strongly respond to parasite-associated signals (74). This motivates a mechanistic agenda: integrate parasite proteomics with host scRNA/ST to identify parasite-to-host signaling relationships and validate candidates experimentally. A second opportunity is to connect parasite intrinsic programs to host niches. Parasite transcriptome studies that resolve germinative and stem-like cellular programs can identify key pathways involved in growth, immune evasion, and drug tolerance (30). Spatial coupling of parasite programs with host lesion zones could move the field beyond descriptive atlases toward cross-kingdom niche biology.

## 5 Putting cell states back into place: ST maps the “firewall” architecture

Single-cell analyses reveal who is present and what they express, yet HE pathobiology is ultimately organized by where these programs reside along the parasite–host boundary and fibrotic rim. scRNA-seq alone cannot establish spatial context, and this limitation is especially consequential in hepatic echinococcosis, where the lesion is organized as a structured “firewall” comprising parasite-proximal inflammatory zones, an immune-exclusion belt, and a collagen-dense fibrotic barrier.ST provides a direct way to test whether inferred immunoregulatory states truly localize to parasite-facing niches, whether angiogenic programs align with defined vascular corridors, and how stromal modules assemble into a diffusion-limiting rim. In a landmark AE study integrating bulk RNA-seq, scRNA-seq, and high-resolution ST, the authors constructed a spatiotemporal atlas of murine infection foci extending to late stages and proposed that disease progression reflects a shift from “active killing” programs toward an immunosuppressive “negative segregation” phenotype that constrains efficient parasite clearance (70). Critically, the spatial component was not used merely for visualization; it supported quantitative, distance-aware analyses by expanding outward from lesion centers in fixed step sizes and computing both cell-type composition and pathway activities along radial gradients, thereby turning lesion architecture into an explicit analytical axis rather than a qualitative descriptor.

Within this framework, the AE study leveraged ST to resolve myeloid–stromal coupling and to prioritize functionally distinct innate immune states *in situ*. Neutrophils captured on ST chips were re-clustered into two subpopulations, and the authors scored curated functional programs, including phagocytosis, degranulation, NETosis, trogocytosis, and multiple regulated cell-death signatures—highlighting that neutrophil states are spatially patterned and temporally dynamic across lesion development. Consistent with this, orthogonal staining supported spatially restricted neutrophil phenotypes, including IL-1β–associated and MPO-associated programs, which the authors linked to parasite-killing functions such as phagocytosis and NET formation (70). Beyond neutrophils, the study implemented module scoring to quantify Spp1^+^ monocyte-derived macrophage (MoMF) signatures and fibroblast signatures per spatial bin, then performed correlation-based co-localization analyses that nominate myeloid–fibroblast adjacency as a core organizing feature of lesion remodeling. Importantly, ligand–receptor inference was performed in a space-aware manner using CellChat, providing a pragmatic route to elevate communication hypotheses that are anatomically plausible within the lesion “firewall”. Together, these results motivate a zone-aware ST design principle for HE: prioritize distance-to-parasite and barrier geometry as first-class covariates and use ST not only to map cell states but to quantify spatial gradients, co-localization, and proximity-filtered communication that can be tested experimentally.

For CE, where the pericyst and laminated layer impose a particularly strong structural boundary, spatial designs that quantify distances to parasite structures, incorporate matched histology, and use serial sections to integrate RNA maps with collagen and vascular staining should substantially strengthen causal inference. Methodologically, modern ST platforms provide a flexible toolbox to match biological questions to spatial resolution and field-of-view constraints (75–77). Analytically, because many ST technologies measure multi-cellular spots/bins, principled deconvolution and cell-state mapping are increasingly necessary to avoid over-interpreting mixed signals, particularly at lesion edges where myeloid, endothelial, and stromal compartments are tightly interwoven (78). Finally, experience from spatial profiling in fibrotic liver diseases reinforces an HE-relevant point: spatially resolved stromal programs can define discrete micro-niches with distinct immune recruitment and barrier properties, arguing that “where” a fibrotic program occurs can be as informative as “whether” it is upregulated in bulk (79).

## 6 Challenges and future directions

HE imposes technical and analytical constraints that are often underestimated and can systematically distort biological inference. Fibrotic rims and necrotic cores introduce strong dissociation bias, meaning that the resulting “cellular census” may reflect enzyme choice and mechanical force as much as genuine tissue composition. In parallel, cyst fluid and necrotic debris elevate ambient RNA and increase the risk that lesion-adjacent hepatocyte transcripts are spuriously detected in immune clusters unless decontamination is explicitly audited. Importantly, under-representation of parenchymal compartments is not merely a missing-cell-type problem; it can actively reshape downstream conclusions by biasing network reconstruction. If fragile hepatocytes and cholangiocytes are selectively lost, ligand–receptor analyses will preferentially highlight immune–immune loops while undercalling parenchymal–immune signaling that may in fact govern nutrient availability, bile acid cues, and immunometabolic control at the lesion edge. Cross-study variability further compounds these issues because lesion stage and sampling distance are frequently reported loosely, even though either variable can invert the apparent direction of key signals. In HE, distance-to-parasite behaves as a latent confounder that can masquerade as a subtype effect, particularly when AE datasets remain relatively scarce and heterogeneous compared with CE, making “CE versus AE” contrasts vulnerable to over-interpretation when sampling zones, chronicity, or treatment status are not tightly controlled.

To guide the next phase of mechanistic and translational work, we propose three integrative concepts. First, lesion-edge zonation should be treated as the organizing principle for study design and interpretation, analogous to the tumor invasive front. Operationally defining a parasite interface, an immune-exclusion myeloid belt, a fibrotic barrier, and distal liver would standardize sampling, enable meaningful cross-cohort comparisons, and provide the minimal spatial framework needed to test whether predicted communication truly co-localizes *in situ*. Second, immunosuppression, angiogenesis, and fibrosis should be viewed as a coordinated survival triad rather than independent sequelae. Under this model, Spp1^+^ remodeling macrophages represent a plausible hub state capable of coupling myeloid–endothelial and myeloid–fibroblast relays, predicting that targeting a single arm may be insufficient unless the coupling points that sustain the triad are also weakened. Third, chronic hepatic lesions may imprint peripheral immune states and circulating small RNAs, enabling viability- and relapse-oriented monitoring. A pragmatic strategy involves first defining lesion-state signatures in tissue through scRNA-seq and spatial profiling, then mapping these signatures to corresponding peripheral correlates measured in PBMC single-cell states and plasma small RNA profiles. These associations should subsequently be validated against clinical imaging stages and treatment responses. This approach shifts the focus from binary presence or absence biomarkers toward dynamic, activity-aware monitoring that reflects parasite viability and disease progression Together, these concepts also imply a stage-aware therapeutic logic: early disease may benefit from boosting effective antigen presentation and parasite killing while avoiding excessive immunopathology, whereas late disease may require dismantling remodeling states and improving drug penetration through antifibrotic or vascular-normalizing adjuncts; checkpoint-like immunomodulation remains conceptually attractive but high-risk and should be evaluated only with stringent stage stratification and safety endpoints.

In addition, to mitigate these HE-specific analytical pitfalls and improve cross-study comparability, we developed HEscTools, a R toolkit tailored for hepatic echinococcosis scRNA-seq. HEscTools provides an integrated workflow for (i) QC auditing with explicit checks for hepatocyte-marker leakage and ambient RNA risk, (ii) metadata validation and standardization of lesion-context variables (e.g., stage, lesion-zone labels, and distance-to-parasite when available), and (iii) disease-relevant module scoring focused on remodeling, fibrosis, and T-cell dysfunction programs, together with lightweight visualization outputs for rapid reporting. By formalizing lesion context as first-class metadata and packaging common HE quality diagnostics into reusable functions, HEscTools bridges the gap between conceptual recommendations and executable practice, enabling more reproducible analyses of fibrotic/necrotic lesions and facilitating meta-analytic integration across cohorts.

Future progress will depend on elevating “where the tissue came from” to a first-class experimental variable and replacing convenience sampling with lesion-zone logic. At a minimum, studies should encode operational zone, imaging stage, treatment history, and parasite strain/species in machine-readable metadata and release processed matrices with harmonized annotations to enable reproducibility and meta-analysis rather than isolated case studies. Mechanistically, the field should move beyond correlation by integrating parasite secretome or transcriptome measurements with host cell-state programs and validating predicted hubs and communication axes *in situ* using spatial profiling alongside orthogonal assays such as immunohistochemistry, flow cytometry, and targeted proteomics. Cross-species integration should be treated as a modeling problem: instead of forcing one-to-one cell-type matching, a more robust approach is to align conserved activation modules and remodeling programs using ortholog-aware gene sets and then quantify which components are host-general versus host-specific across human disease, mouse models, and relevant livestock hosts. Ultimately, a community HE cells atlas will be most valuable if it is explicitly One Health in scope and built around lesion-edge zonation, with design choices optimized not only for descriptive completeness but also for translational endpoints such as viability-oriented monitoring and stage-aware intervention strategies.
